# Targeting Spermine Oxidase to Mitigate Traumatic Brain Injury Pathology in the Aging Brain

**DOI:** 10.3390/antiox14060709

**Published:** 2025-06-11

**Authors:** Jui-Ming Sun, Jing-Shiun Jan, Ting-Lin Yen, Yu-Hao Chen, Ruei-Dun Teng, Chih-Hao Yang, Cheng-Ta Hsieh

**Affiliations:** 1 Section of Neurosurgery, Department of Surgery, Ditmanson Medical Foundation, Chia-Yi Christian Hospital, Chia-Yi City 600, Taiwan; 07178@cych.org.tw (J.-M.S.); 07454@cych.org.tw (Y.-H.C.); 2 Department of Nursing, School of Nursing, Fooyin University, Kaohsiung City 831, Taiwan; 3 Department of Pharmacology, School of Medicine, College of Medicine, Taipei Medical University, Taipei 110, Taiwan; d119101004@tmu.edu.tw (J.-S.J.); d119096015@tmu.edu.tw (T.-L.Y.); tang0803@tmu.edu.tw (R.-D.T.); chyang@tmu.edu.tw (C.-H.Y.); 4 Department of Medical Research, Cathay General Hospital, Taipei 106, Taiwan; 5 Chung-Jen Junior College of Nursing, Health Sciences and Management, Chia-Yi City 622, Taiwan; 6 Division of Neurosurgery, Department of Surgery, Cathay General Hospital, Taipei 106, Taiwan; 7 School of Medicine, National Tsing Hua University, Hsinchu 300, Taiwan; 8 Department of Medicine, School of Medicine, Fu Jen Catholic University, New Taipei City 242, Taiwan

**Keywords:** spermine oxidase, traumatic brain injury, aging brain, oxidative stress, neuro-inflammation, neuronal cell death, brain injury therapeutics

## Abstract

Traumatic brain injury (TBI) in the elderly is frequently associated with worsened neurological outcomes and prolonged recovery, yet the age-specific molecular mechanisms driving this vulnerability remain poorly understood. Aging is characterized by increased oxidative stress and chronic neuro-inflammation, both of which may amplify the brain’s susceptibility to injury. In this study, we identify spermine oxidase (SMOX), a polyamine-catabolizing enzyme that produces reactive oxygen species, as a key mediator linking oxidative stress and neuro-inflammation to age-dependent TBI susceptibility. Using a mouse model of controlled cortical impact (CCI), we found that SMOX expression was significantly upregulated in aged brains, primarily in neurons and microglia, and this increase correlated with greater microglial activation, elevated pro-inflammatory cytokine expression, and widespread neuronal degeneration. Notably, SMOX upregulation also impaired astrocytic glutamate clearance by disrupting the membrane localization of the transporter GLT-1, contributing to excitotoxic stress. Importantly, analysis of postmortem human brain samples and transcriptomic data revealed a parallel age-related increase in SMOX expression, supporting its translational relevance. The pharmacological inhibition of SMOX with JNJ-9350 in aged mice reduced oxidative and inflammatory markers, preserved neuronal viability, and improved motor, cognitive, and emotional outcomes up to 30 days post-injury. These findings establish SMOX as a critical molecular driver of age-related vulnerability to TBI and highlight its inhibition as a promising therapeutic strategy for improving outcomes in elderly TBI patients.

## 1. Introduction

Traumatic brain injury (TBI) is a leading cause of long-term disability and mortality worldwide, with disproportionately severe impacts on elderly populations [1,2,3]. Older individuals exhibit poorer recovery trajectories and a higher incidence of neurological complications compared to younger patients [4]. As the global population ages, the proportion of TBI cases occurring in elderly individuals continues to rise, representing a growing public health challenge [5,6]. Clinical data consistently demonstrate that aging is a critical determinant of poor TBI outcomes, with aged individuals exhibiting exacerbated neurological deficits, increased lesion volumes, and greater susceptibility to post-traumatic complications, such as chronic neuro-inflammation and dementia [7,8,9]. Despite this, the mechanistic underpinnings of age-associated vulnerability to TBI remain incompletely understood, and most therapeutic strategies are still developed based on data derived from young adult models. This knowledge gap limits the development of age-specific therapeutic strategies and represents a critical barrier to improving clinical care for this vulnerable population.

One of the prominent hallmarks of aging is the progressive accumulation of oxidative stress in brain tissue [10]. With advancing age, the capacity of endogenous anti-oxidant systems declines, while reactive oxygen species (ROS) and other free radicals accumulate, damaging cellular macromolecules and impairing mitochondrial function [11,12]. Following TBI, a secondary wave of oxidative injury occurs, amplifying neuronal death, blood–brain barrier disruption, and neuro-inflammatory cascades. Importantly, studies have shown that the oxidative stress burden following TBI is more pronounced in aged brains, where the imbalance between pro-oxidant and anti-oxidant systems is already tipped toward vulnerability [13,14]. Despite growing interest in redox-targeted interventions, most anti-oxidant therapies have yielded limited success in clinical trials, underscoring the need to identify upstream enzymatic drivers of oxidative damage that are specifically active in aged or injured brains.

Among these potential drivers is spermine oxidase (SMOX), a flavin-dependent enzyme involved in polyamine catabolism [15,16]. SMOX catalyzes the back-conversion of spermine into spermidine, generating hydrogen peroxide as a byproduct. This ROS-generating property positions SMOX as a critical regulator of redox homeostasis, particularly under pathological conditions [17]. Although SMOX is typically expressed at low levels in the brain, its expression is upregulated in response to injury, ischemia, and neuro-inflammatory stimuli. In models of Alzheimer’s disease, Parkinson’s disease, and cerebral ischemia, SMOX has been shown to promote neuronal injury via oxidative stress and mitochondrial dysfunction [17,18,19]. In the context of traumatic brain injury (TBI), prior work by Zahedi and colleagues demonstrated that SMOX is significantly upregulated in both neurons and glial cells between 3 and 7 days post-injury, highlighting its role in driving secondary damage through oxidative stress [20]. However, the interplay between SMOX expression and aging has not been clearly defined. Whether the age-associated increase in SMOX expression alters the brain’s susceptibility to injury or interacts with the acute SMOX response to trauma remains unknown. This raises the critical question of whether SMOX is merely a downstream consequence of TBI or a predisposing factor that contributes to worsened outcomes in aged brains. Addressing this gap has important therapeutic implications for age-tailored interventions in TBI.

In this study, we investigated the role of SMOX in mediating age-dependent susceptibility to TBI. Using a controlled cortical impact (CCI) mouse model, we performed immunohistochemistry, molecular profiling, and behavioral assessments to examine the cell-type-specific expression of SMOX in aged brains, its association with oxidative stress and inflammation, and its contribution to neuronal death. We further evaluated the therapeutic potential of JNJ-9350, a potent and selective SMOX inhibitor, in mitigating both acute and long-term TBI-related pathologies. Our findings identify SMOX as a central mediator of age-associated oxidative stress and neuro-inflammation following TBI and support its inhibition as a promising therapeutic strategy for improving outcomes in this growing high-risk population.

## 2. Materials and Methods

### 2.1. Animals

Male C57BL/6 mice were used in this study and categorized into three age groups: young (9–12 weeks), middle-aged (36–40 weeks), and aged (>60 weeks). All animals (25–30 g) were obtained from BioLASCO (Taipei, Taiwan). Upon arrival, mice were verified to be in normal health, free from pathogens, and without any signs of neurological impairment. A 7-day acclimatization period was provided before initiating any experimental procedures. Mice were housed in a conventional pathogen-free facility under standard temperature and humidity conditions with a 12 h light/dark cycle (lights on at 07:00, off at 19:00), and food and water were provided ad libitum. All procedures were conducted in accordance with institutional guidelines and approved by the Institutional Animal Care and Use Committee of Cathay General Hospital (Approval No. CGH-IACUC-113-021; approval period: 1 July 2024 to 30 June 2026).

### 2.2. Traumatic Brain Injury Model

To induce traumatic brain injury, a CCI paradigm was applied using a precision impactor system based on a previously validated protocol with slight adjustments [13,21]. Mice were anesthetized with 4% vol. isoflurane for induction and secured in a stereotaxic frame. After a midline scalp incision, a 3.5 mm circular craniotomy was performed over the right hemisphere. The craniotomy site was centered 2 mm lateral to the sagittal suture and 2 mm posterior to the bregma, allowing exposure of the intact dura mater.

The exposed cortical surface was then subjected to a mechanical impact using a 3 mm flat-tip piston, delivered at a speed of 5.0 m/s, with an impact depth of 1.0 mm and a dwell time of 300 milliseconds. Following the injury, the scalp was carefully sutured, and mice were placed on a heated pad to recover before being returned to their home cages.

### 2.3. Behavioral Testing Procedures

A comprehensive battery of behavioral tests was conducted to assess motor, cognitive, and emotional functions in mice following traumatic brain injury. All tests were performed during the light phase under consistent environmental conditions, and mice were habituated to the testing room for at least 30 min prior to each session. Assessments were conducted at both early (day 2 or 7) and chronic (day 30) post-injury time points, as specified in each experiment.

### 2.4. Neurological Severity Score (NSS)

Neurological function was assessed using an 18-point sliding scale [22], where a score of 0 indicates normal function and a score of 18 reflects maximal neurological impairment. The evaluation included assessments of reflexes, motor ability, balance, and coordination through a standardized series of tasks. Higher scores corresponded to greater degrees of functional deficit following injury.

### 2.5. Grip Strength Test

Forelimb grip strength was measured using a digital grip strength meter (UGO Basile, Varese, Italy) to assess neuromuscular function following CCI. Mice were allowed to grasp a horizontal bar with their forepaws and were gently pulled backward until they released. The peak force generated by the forelimbs was recorded in gram-force (gf). Each mouse underwent three consecutive trials, and the average of the three measurements was used for analysis. Testing was performed at baseline (pre-injury) and again at two and seven days post-CCI.

### 2.6. Y-Maze Spontaneous Alternation Test

The Y-maze apparatus consisted of three arms positioned at 120° angles. Mice were placed at the end of one arm and allowed to explore freely in the three arms for ten minutes. The sequence and number of arm entries were recorded. Spontaneous alternation was defined as successive entries into all three arms without repetition. The percentage of spontaneous alternations was calculated as an index of spatial working memory.

### 2.7. Novel Object Recognition Memory (ORM) Test

The ORM test was used to assess recognition memory following TBI. The task was conducted over two consecutive days in an open-field arena. On day 1 (habituation), mice were allowed to explore the empty arena for 10 min. On day 2, mice first underwent a familiarization phase. During this, two identical objects were placed in the arena and explored freely by mice for 10 min. After a 1 h retention interval, one of the familiar objects was replaced with a novel object of similar size but different shape and texture. Mice were reintroduced to the arena and allowed to explore for 5 min. Object exploration was recorded, and recognition memory was expressed as a preference percentage: Novel Object Preference (%) = [Time spent exploring the novel object/Total object exploration time] × 100.

### 2.8. Open-Field Test

The open-field test was used to assess general locomotor activity and anxiety-like behavior. Mice were individually placed in the center of a square open-field arena (45 × 45 cm) and allowed to explore freely for 10 min. Animal movement was recorded and analyzed using automated video tracking software known as Ethovision XT 17 (Noldus). Two primary behavioral parameters were measured: total distance traveled (as an indicator of locomotor activity) and time spent in the central zone versus the periphery (as an index of anxiety-like behavior). Reduced exploration of the central area was interpreted as increased anxiety-like behavior.

### 2.9. Novelty-Suppressed Feeding (NSF) Test

The NSF test was used to assess anxiety-like behavior in a conflict-based setting. Mice were food-deprived for 24 h prior to testing. On the test day, each mouse was placed in a novel open-field arena with a single food pellet positioned at the center. The latency to approach and begin feeding was recorded during a 10 min observation period. Immediately after the test, mice were returned to their home cages, and latency to feed was recorded again to confirm normal feeding motivation. A longer latency to initiate feeding in the novel environment was interpreted as an anxiety-like behavioral response.

### 2.10. Drug Treatment

JNJ-9350, a selective spermine oxidase (SMOX) inhibitor [23], was used to evaluate the therapeutic potential of SMOX suppression in traumatic brain injury. The initial dosing strategy was guided by the NIH Guidance Document on Using In Vitro Data to Estimate In Vivo Starting Doses for Acute Toxicity (NIH Publication No. 01-4500). Based on this guideline, two preliminary doses—10 mg/kg and 5 mg/kg—were selected for in vivo evaluation.

Initial screening revealed that both doses produced comparable efficacy in reducing brain oxidative stress levels post-TBI. Given the similar biological effects and to minimize potential off-target toxicity, the lower dose of 5 mg/kg was selected for use in all subsequent experiments.

JNJ-9350 was dissolved in a vehicle consisting of 10% DMSO, 10% Cremophor EL, 5% Tween-80, and 75% sterile normal saline. The compound remained fully soluble without visible precipitation throughout preparation and administration. We administered a drug or vehicle intraperitoneally once daily for three consecutive days following CCI surgery.

### 2.11. Quantification of Malondialdehyde (MDA) and Hydroxyl Free Radicals

Malondialdehyde (MDA), a reactive three-carbon dialdehyde generated during polyunsaturated fatty acid peroxidation, was measured as a biomarker of lipid peroxidation and oxidative stress. Approximately 25 mg of injured brain tissue from each mouse was homogenized in the assay buffer and centrifuged at 1600× *g* for 10 min at 4 °C. To minimize variability due to the differences in water content across samples, MDA levels were normalized to total protein concentrations, as determined using a bicinchoninic acid (BCA) assay. The resulting supernatants were analyzed using a commercial MDA assay kit (Cat. No. 10009055, Cayman Chemical, Ann Arbor, MI, USA) according to the manufacturer’s instructions. Absorbance was measured at 535 nm using a microplate reader, and MDA concentrations were calculated based on a standard curve.

Hydroxyl free radical levels were assessed using a colorimetric hydroxyl radical assay kit (Cat. No. MBS2540424, MyBioSource, San Diego, CA, USA). Briefly, freshly isolated brain tissue blocks were processed as per the manufacturer’s protocol, and the formation of hydroxyl radicals was quantified by measuring the absorbance of the reaction product at 550 nm. All readings were performed using a calibrated microplate spectrophotometer.

### 2.12. Histological and Immunofluorescence Analyses

For immunofluorescence experiments, mice were euthanized under deep anesthesia at indicated time points post-CCI or following sham surgery. Mice were transcardially perfused with phosphate-buffered saline (PBS), followed by treatment with 4% paraformaldehyde (PFA) (Sigma-Aldrich, Merck, St. Louis, MO, USA). Brains were then extracted and post-fixed in 4% PFA overnight at 4 °C, followed by cryoprotection in 30% sucrose at 4 °C for 72 h or until the tissue sank. Coronal brain sections (50 μm thick) were obtained using a sliding microtome (Leica SM2010R, Germany) and processed for immunofluorescent staining.

In parallel, postmortem human frontal cortical brain sections from adult (<45 years) and aged (>75 years) individuals were obtained from the BioChain Institute (Newark, CA, USA). These paraformaldehyde-fixed cryosections were used to evaluate SMOX expression and age-associated cellular changes. All procedures involving human tissues complied with institutional ethical guidelines and were performed according to the supplier’s protocols.

For immunostaining, free-floating sections were first incubated for 1 h in a blocking solution containing donkey serum and 0.3% Triton X-100 (Sigma-Aldrich, Merck, St. Louis, MO, USA). This was followed by overnight incubation at 4 °C with a mixture of primary antibodies. The following primary antibodies and concentrations were used: SMOX (Thermo Fisher Scientific, Waltham, MA, USA, PA5-100112, 1:500), NeuN (Sigma-Aldrich, St. Louis, MO, USA, ABN91, 1:2000), Iba1 (Wako Chemicals, Richmond, VA, USA, 019-19741, 1:2000), GFAP (Abcam, Cambridge, MA, USA, ab4674, 1:1000), 4-hydroxy-2E-nonenal (4-HNE; Abcam, ab46545, 1:500), 8-oxo-2′-deoxyguanosine (8-oxo-dG; JaICA, MOG-100P, 1:200), and CD31 (Abcam, ab28364, 1:1000). The next day, sections were incubated overnight at 4 °C with species-appropriate secondary antibodies conjugated to CF488A, CF568, or CF633 fluorophores (Biotium, Fremont, CA, USA). To minimize the background interference caused by lipofuscin autofluorescence, especially in aged brain sections, samples were treated with TrueBlack^®^ Lipofuscin Autofluorescence Quencher (Biotium) according to the manufacturer’s instructions.

Following three washes with PBS containing 0.05% Tween-20, sections were mounted using ProLong™ Glass Antifade Mountant (Thermo Fisher Scientific) and cover-slipped. Fluorescent images were acquired using a Leica STELLARIS 8 confocal microscope at 40× magnification. Quantification was performed manually in either the prefrontal cortex or hippocampal CA1 regions. For each image, three regions of interest (50 × 50 μm^2^) were selected, and the number of positively stained cells was normalized and presented as cells per mm^2^.

### 2.13. RNA Extraction and Quantitative Real-Time PCR

For gene expression analysis, mice were euthanized at indicated time points following sham or CCI surgery. Mice were anesthetized and perfused with ice-cold PBS, after which cortical and hippocampal tissues were rapidly dissected, flash-frozen in liquid nitrogen, and homogenized. Total RNA was extracted using the NucleoSpin RNA extraction kit (Macherey-Nagel, Düren, Germany) according to the manufacturer’s protocol. RNA concentrations were determined using a NanoDrop spectrophotometer, and 2 µg of RNA from each sample was reverse-transcribed into cDNA using the SuperScript™ IV First-Strand Synthesis System (Thermo Fisher Scientific, Waltham, MA, USA).

Quantitative real-time PCR was performed using the QuantiNova SYBR Green PCR Kit (Qiagen, Hilden, Germany) on an Applied Biosystems™ Real-Time PCR System (Thermo Fisher Scientific). Gene-specific primer sequences used for amplification are listed in Table 1. Relative mRNA expression levels were calculated using the ΔΔCt method, with GAPDH serving as the internal reference gene.

### 2.14. Statistical Analysis

All data are presented as mean ± standard error of the mean (SEM). Statistical comparisons between multiple groups were performed using one-way analysis of variance (ANOVA), followed by the use of Bonferroni’s post hoc test to correct for multiple comparisons. For two-group comparisons, unpaired two-tailed Student’s *t*-tests were used where appropriate. Correlation analyses were conducted using Pearson’s correlation coefficient.

A *p* value of less than 0.05 was considered statistically significant. All statistical analyses were performed using GraphPad Prism software (version 8; GraphPad Software, San Diego, CA, USA). Sample sizes, the number of replicates, and specific statistical tests used for each figure are indicated in the figure legends.

## 3. Results

### 3.1. Middle-Aged Mice Exhibit Impaired Functional Recovery Following TBI, Associated with Exacerbated Oxidative Stress Responses

**Figure 1:**
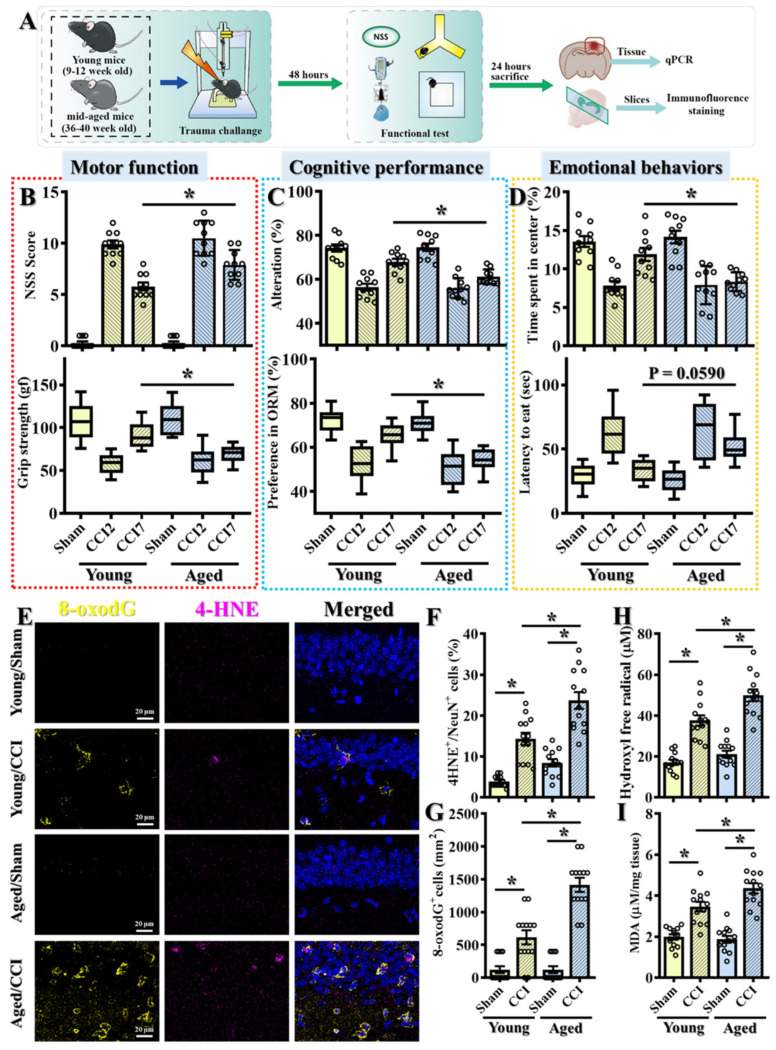
Increased oxidative stress correlates with exacerbated TBI phenotypes in aged mice. (**A**) Schematic overview of experimental design used to compare TBI outcomes between young and middle-aged mice following CCI. (**B**) Quantification of motor function impairments assessed by Neurological Severity Score (NSS) and grip strength tests in mice with or without CCI. (**C**) Cognitive performance evaluated by spontaneous alternation in Y-maze and novel object recognition memory (ORM) test. (**D**) Emotional behavior analysis based on open-field center zone entries and latency to feed in novelty-suppressed feeding (NSF) test. (**E**–**G**) Representative immunofluorescence images and quantification of oxidative stress markers 4-hydroxy-2E-nonenal (4-HNE, magenta) and 8-oxo-2′-deoxyguanosine (8-oxo-dG, yellow) in hippocampal CA1 region across experimental groups. Nuclei are counterstained with DAPI (blue). Scale bar = 20 μm. Quantification based on 12 images per group (n = 4 mice/group). (H–I) Biochemical quantification of hydroxyl free radical production (**H**) and malondialdehyde (MDA) concentrations (**I**) in cortical tissues from different experimental groups. Small circles in F–I represent individual data points from biological replicates. All data are presented as mean ± SEM and analyzed by one-way ANOVA followed by Bonferroni’s post hoc test. * *p* < 0.05.

Since most therapeutic strategies for TBI have been developed using young adult models [24,25], we aimed to develop age-targeted interventions by focusing on older populations. However, very aged mice often exhibit baseline impairments in motor, cognitive, and emotional functions, which can confound the interpretation of TBI-induced changes [13]. Therefore, we selected middle-aged mice (36–40 weeks old) for this study and compared their responses to those of young adult mice (9–12 weeks old) (Figure 1A). Both groups were subjected to controlled cortical impact (CCI), and behavioral assessments were conducted at two time points post-injury: day 2 (CCI2) and day 7 (CCI7).

At the early time point (CCI2), young adult and middle-aged mice exhibited comparable levels of impairment across multiple behavioral domains. These included motor function (Figure 1B), assessed using the Neurological Severity Score (NSS) and grip strength tests; cognitive performance (Figure 1C), evaluated by the Y-maze spontaneous alternation test and the novel object recognition (NOR) test; and anxiety-like emotional behaviors (Figure 1D), assessed through open-field exploration and the novelty-suppressed feeding (NSF) test. These findings suggest that both age groups experience similar levels of initial traumatic damage, allowing for a more accurate comparison of their recovery capacities over time.

However, over time, recovery trajectories diverged significantly between the two age groups. By seven days post-injury (CCI7), young adult mice exhibited substantial improvement across all behavioral domains, while middle-aged mice showed persistent deficits, particularly in motor coordination, cognitive performance, and anxiety-like behaviors (Figure 1B–D). These findings indicate that although the initial injury severity was comparable, the aged brain demonstrates reduced resilience and impaired recovery following TBI.

Given the well-established role of oxidative stress in TBI pathophysiology [26,27,28], we next investigated whether age-related differences in oxidative stress responses could underlie the observed disparities in recovery. 4-HNE is a reactive aldehyde generated during lipid peroxidation and serves as a reliable marker of oxidative lipid damage [29]. Similarly, 8-oxo-2′-deoxyguanosine (8-oxo-dG) is a widely recognized marker of oxidative DNA damage and is commonly used to assess DNA oxidation following oxidative stress [30]. Both markers have been extensively validated in TBI and neurodegeneration studies.

Immunohistochemical analysis revealed a significantly higher number of 4-HNE-positive neurons (Figure 1F; Young/CCI: 14.31 ± 1.491% vs. Aged/CCI: 23.69 ± 2.058%, *p* < 0.01) and 8-oxo-dG-positive cells (Figure 1G; Young/CCI: 615.4 ± 107.3 cells/mm^2^ vs. Aged/CCI: 1415 ± 96.49 cells/mm^2^, *p* < 0.01) in the peri-contusional cortex of middle-aged mice compared to young adults. These results indicate the significantly elevated lipid peroxidation and oxidative DNA damage in the aged brain following TBI, suggesting an exacerbated oxidative stress response as a potential mechanism for impaired recovery.

To further validate the enhanced oxidative stress response in the aged brain, we also performed biochemical analyses to quantify hydroxyl free radicals and malondialdehyde (MDA) levels—both established indicators of oxidative damage. Hydroxyl radicals are among the most reactive species generated during oxidative stress, while MDA is a well-characterized byproduct of lipid peroxidation. Consistent with our immunohistochemical findings, middle-aged TBI mice exhibited significantly elevated levels of hydroxyl free radicals (Figure 1H) and increased MDA concentrations (Figure 1I) in cortical tissues compared to young adult TBI mice, confirming the heightened redox imbalance in the aged brain following injury.

Collectively, these findings suggest that exaggerated oxidative stress responses are closely associated with the pathological mechanisms underlying worsened functional outcomes in aged mice following TBI. The persistence of oxidative injury in the aged brain likely compromises neuronal survival and interferes with tissue repair processes, thereby contributing to the impaired recovery and heightened TBI susceptibility observed with aging.

### 3.2. Age-Dependent Increase in SMOX Expression Correlates with Oxidative Stress and Behavioral Vulnerability to TBI

**Figure 2:**
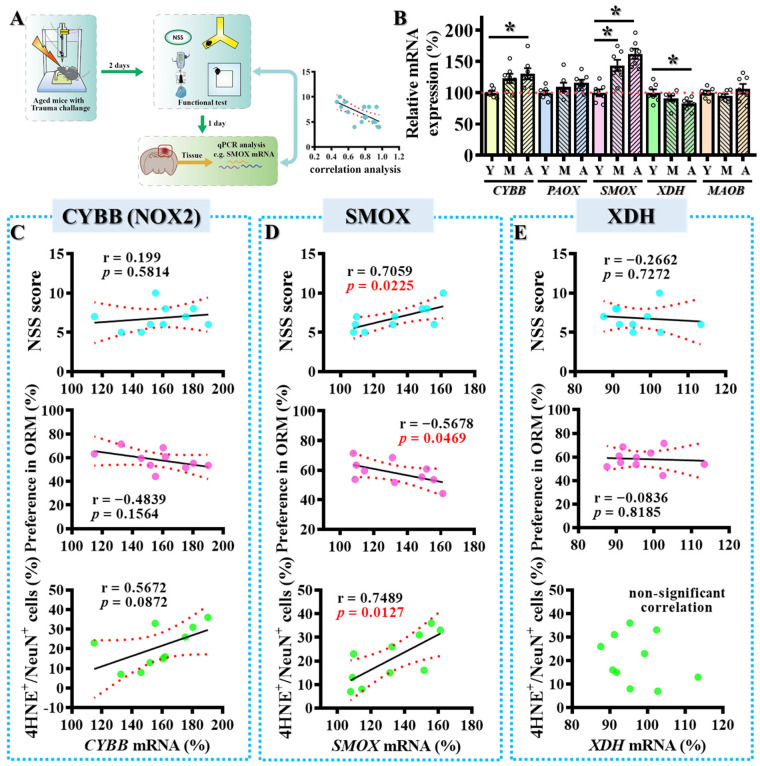
Age-dependent increase in SMOX expression correlates with oxidative stress and behavioral vulnerability following TBI. (**A**) Schematic overview of experimental design used to assess correlations between gene expression levels and individual behavioral or histological outcomes post-TBI. (**B**) Quantitative analysis of mRNA expression levels of enzymatic regulators of hydroxyl radical production in cortical tissues from young (Y), middle-aged (M), and aged (A) mice. Data are presented as mean ± SEM and analyzed by one-way ANOVA followed by Bonferroni’s post hoc test. * *p* < 0.05. (**C**–**E**) Pearson’s correlation analyses between relative expression levels of Cybb (NOX2) (**C**), Smox (**D**), and Xdh (**E**) with individual TBI outcomes, including motor function (NSS), cognitive performance (ORM), and oxidative stress marker levels (4-HNE-positive cells). n = 10 mice. Small circles represent individual biological replicates. Different colors correspond to distinct experimental groups or treatment conditions.

Having observed elevated oxidative stress in middle-aged mice following TBI, we next investigated whether age-dependent transcriptional changes in key enzymatic regulators of hydroxyl radical production could underlie this phenotype. We focused on a panel of enzymes known to be enriched in the brain and implicated in the metabolism and generation of hydroxyl free radicals [31,32,33], including NADPH oxidase 2 (NOX2), polyamine oxidase (PAOX), spermine oxidase (SMOX), xanthine dehydrogenase (XDH), and monoamine oxidase B (MAOB) (Figure 2A,B).

Using quantitative PCR analysis of cortical brain lysates, we compared the expression of these genes across three age groups: young (9–12 weeks), middle-aged (36–40 weeks), and aged (>60 weeks). Among the five candidates, SMOX displayed a robust and significant age-dependent increase in expression, with transcript levels markedly elevated in both middle-aged and aged mice compared to young controls (Figure 2B; young: 100 ± 5.546%, middle-aged: 143.7 ± 9.002%, aged: 162.3 ± 8.094%; *p* < 0.01 vs. young group). In contrast, CYBB (which encodes the NOX2 protein) showed a mild but non-significant increase with age, while XDH expression was significantly reduced in the aged group. PAOX and MAOB levels remained relatively unchanged across the three age groups (Figure 2B).

To further assess the potential pathological relevance of SMOX upregulation, we conducted correlation analyses between the expression levels of SMOX, NOX2, and XDH with TBI-related phenotypes in a cohort of CCI-injured mice (Figure 2A,C–E). Strikingly, SMOX mRNA levels exhibited strong correlations with both motor deficits (as measured by NSS) and cognitive impairments (assessed by novel object recognition tasks). Moreover, SMOX expression also correlated significantly with oxidative stress markers, including biochemical measures and the density of 4-HNE immunostaining in cortical regions post-injury. In contrast, no significant correlations were observed for NOX2 or XDH expression with either behavioral deficits or oxidative stress markers (Figure 2C–E).

These findings strongly support a model in which the age-associated upregulation of SMOX contributes to the heightened vulnerability of the aged brain to TBI, likely through the amplification of oxidative stress responses. Among the hydroxyl radical-generating enzymes examined, SMOX emerged as the most dynamically regulated with age and the most strongly correlated with injury-associated outcomes, highlighting its potential as a key pathological driver and therapeutic target in age-related TBI.

### 3.3. SMOX Expression Is Pathologically Elevated in the Aged Brain Across Species, Independent of Injury

**Figure 3:**
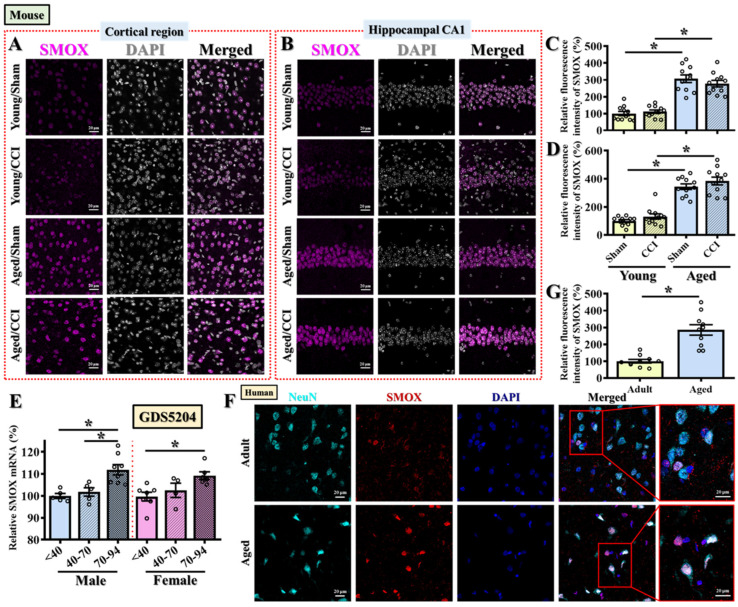
SMOX expression is elevated in aged mouse and human brains. (**A**,**B**) Representative immunofluorescence images, showing SMOX expression (magenta) in cortical region (**A**) and hippocampal CA1 region (**B**) of young and aged mice with or without CCI surgery. Nuclei were counterstained with DAPI (white). Scale bar = 20 μm. (**C**,**D**) Quantification of SMOX fluorescence intensity in cortex (**C**) and hippocampal CA1 region (**D**) across experimental groups. (**E**) Quantitative analysis of SMOX mRNA levels in human frontal cortex samples across different age groups using publicly available GEO dataset GDS5204. (**F**,**G**) Representative immunofluorescence images (**F**) and quantification (**G**) of SMOX (red) and NeuN (cyan) co-staining in postmortem human frontal cortical tissue from young and aged individuals. Nuclei were counterstained with DAPI (blue). Scale bar = 20 μm. Small circles in C–E and G represent individual data points from biological replicates. All data are presented as mean ± SEM and were analyzed using one-way ANOVA followed by Bonferroni’s post hoc test. * *p* < 0.05.

To validate and extend our transcript-level findings from Figure 2, we next examined age-related changes in SMOX protein expression in both mouse and human brains. The immunofluorescent staining of mouse brain sections revealed that, even in the absence of injury, aged mice exhibited significantly elevated SMOX protein expression levels in both the prefrontal cortical regions (Figure 3A,C) and the CA1 subfield of the hippocampus (Figure 3B,D) when compared to young adult controls. Interestingly, CCI surgery did not further enhance SMOX protein expression in aged mice (Figure 3A–D), suggesting that the age-associated upregulation of SMOX is constitutive and may represent a predisposing factor rather than a consequence of injury. To further assess whether injury-induced SMOX changes could still occur in young mice and to spatially map this response across regions proximal to the injury site, we performed additional immunostaining in the somatosensory and motor cortices. As shown in Supplementary Figure S1, SMOX expression was significantly increased in these regions in young adult mice at 3 days post-CCI (Supplementary Figure S1A–D), which is consistent with previous reports of SMOX induction following TBI [20]. In contrast, aged mice showed a markedly elevated basal level of SMOX expression in the same cortical regions, which did not increase further in response to injury. These data suggest that while SMOX is inducible by TBI in young brains, its constitutive elevation in aged brains may saturate this pathway or reflect fundamentally altered regulatory control.

These findings indicate that the elevated SMOX expression observed in aged mice is likely a pathological baseline feature that could contribute to the heightened oxidative stress responses and increased behavioral vulnerability following TBI, as demonstrated in our earlier experiments.

To explore whether this age-dependent SMOX upregulation is also conserved in humans, we first analyzed publicly available transcriptomic data from GDS5204, which includes postmortem frontal cortex samples from neurotypical individuals spanning young (≤40 years), middle-aged (41–70 years), and aged (≥70 years) groups. Consistent with our findings in mice, SMOX mRNA expression was significantly increased with age in both male and female samples (Figure 3E). To validate this at the protein level, we performed immunofluorescent staining on postmortem frontal cortical brain sections taken from adult (<45 years) and aged (>75 years) individuals. SMOX protein expression was markedly elevated in aged human brains (Figure 3F,G; Adult: 100 ± 10.62%, Aged: 285.6 ± 31.03%; *p* < 0.01), closely mirroring the expression pattern observed in mice.

Together, these cross-species findings strongly support the notion that SMOX is an age-associated molecular factor, elevated independently of injury, and may represent a key upstream contributor to the oxidative stress burden and heightened TBI susceptibility observed in older individuals.

### 3.4. Pharmacological Inhibition of SMOX Attenuates Oxidative Stress in the Aged Brain Following TBI

**Figure 4:**
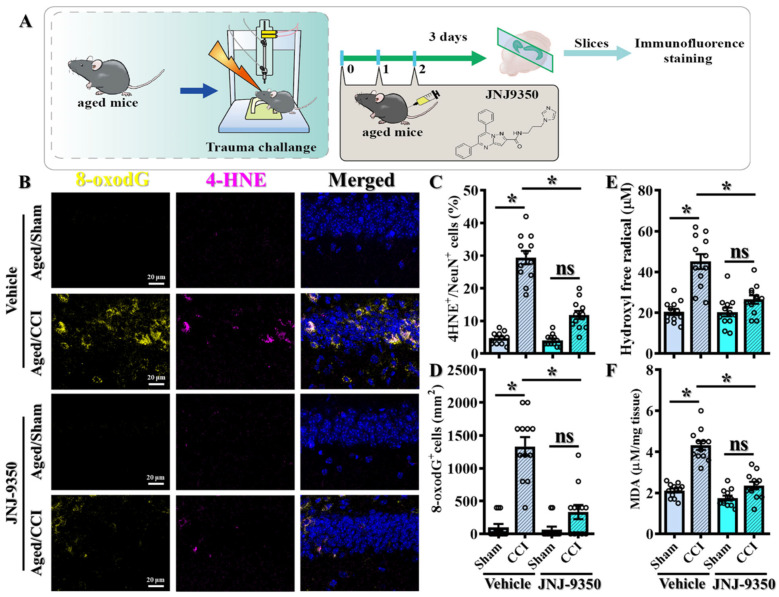
Pharmacological inhibition of SMOX reduces oxidative stress in aged brain following TBI. (**A**) Schematic overview of experimental design used to evaluate effects of SMOX inhibition on oxidative stress in aged mice after CCI. (**B**) Representative immunofluorescence images of oxidative stress markers 4-HNE (magenta) and 8-oxo-2′-deoxyguanosine (8-oxo-dG, yellow) in hippocampal CA1 region. Nuclei are counterstained with DAPI (blue). Scale bar = 20 μm. (**C**,**D**) Quantification of 4-HNE-positive neurons (**C**) and 8-oxo-dG-positive cells (**D**) across experimental groups. Quantification based on 12 images per group (n = 4 mice/group). (**E**,**F**) Biochemical quantification of hydroxyl free radical production (**E**) and malondialdehyde (MDA) concentrations (**F**) in cortical tissues from aged mice following CCI with or without SMOX inhibition. Small circles in C–F represent individual data points from biological replicates. All data are presented as mean ± SEM and were analyzed using one-way ANOVA followed by Bonferroni’s post hoc test. * *p* < 0.05; ns, not significant.

Given the pathological elevation of SMOX expression in the aged brain, we next sought to determine whether inhibiting SMOX activity could functionally mitigate the exaggerated oxidative stress responses observed following TBI. To this end, we employed JNJ-9350, a recently developed and potent SMOX inhibitor [23] that is administered intraperitoneally at 5 mg/kg in aged mice (Figure 4A).

To assess the impact of SMOX inhibition on oxidative damage, we performed immunofluorescent staining for 4-HNE and 8-oxo-2′-deoxyguanosine (8-oxo-dG) at three days post-CCI. 4-hydroxy-2E-nonenal (4-HNE), a reactive aldehyde derived from ω-6 polyunsaturated fatty acid peroxidation, was used as a marker of lipid peroxidation. In parallel, 8-hydroxy-2′-deoxyguanosine (8-oxo-dG) was used to assess oxidative DNA damage. These are well-established markers of lipid peroxidation and oxidative DNA damage, respectively. Notably, treatment with JNJ-9350 significantly reduced the levels of both 4-HNE-positive neurons (Figure 4B,C; JNJ-9350/CCI: 11.83 ± 1.254% vs. vehicle/CCI: 29.42 ± 2.039%, *p* < 0.01) and 8-oxo-dG-positive cells (Figure 4B,D; JNJ-9350/CCI: 333.3 ± 108.2 cells/mm^2^ vs. vehicle/CCI: 1333 ± 142.1 cells/mm^2^, *p* < 0.01) in the brains of aged mice following TBI compared to vehicle-treated controls.

Biochemical analyses further corroborated these findings. The quantification of hydroxyl free radical production and malondialdehyde (MDA) concentrations demonstrated that SMOX inhibition by JNJ-9350 significantly suppressed the overproduction of reactive oxygen species (Figure 4E) and reduced lipid peroxidation (Figure 4F) in the aged brain following TBI.

Collectively, these results demonstrate that SMOX activity is a key driver of the heightened oxidative stress responses observed in aged individuals after brain injury. Importantly, the pharmacological inhibition of SMOX effectively attenuates these pathological oxidative events, supporting the therapeutic potential of SMOX blockade in mitigating TBI-induced damage in the aging brain.

### 3.5. SMOX Inhibition Ameliorates Motor, Cognitive, and Emotional Deficits Following TBI in Aged Mice

**Figure 5:**
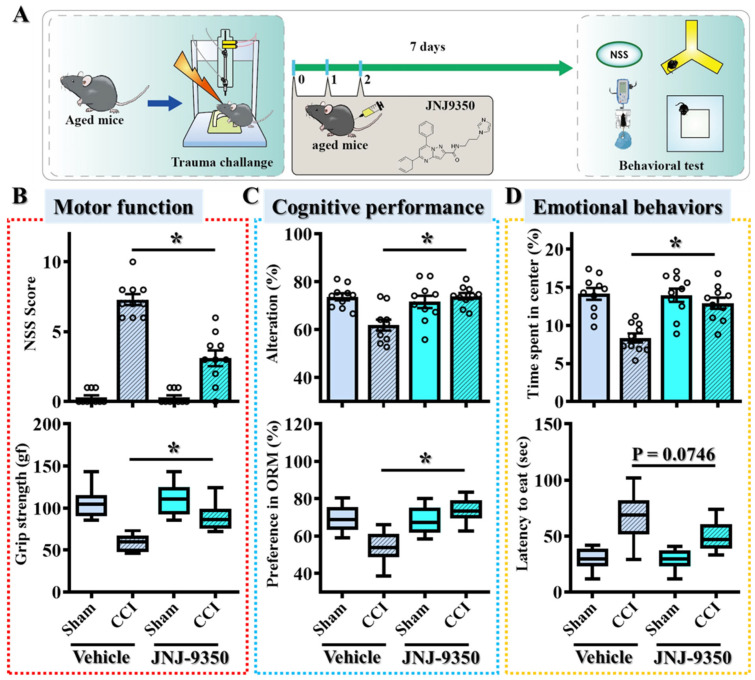
Pharmacological inhibition of SMOX attenuates TBI-induced behavioral deficits in aged mice. (**A**) Schematic overview of experimental design used to assess effects of SMOX inhibition on functional outcomes in aged mice following CCI. (**B**) Quantification of motor function impairments, measured by Neurological Severity Score (NSS) and grip strength, in vehicle- and JNJ-9350-treated mice. (**C**) Cognitive performance assessed using Y-maze spontaneous alternation test and novel object recognition memory (ORM) test. (**D**) Emotional behavior evaluated through open-field center zone exploration and latency to feed in novelty-suppressed feeding (NSF) test. All data are presented as mean ± SEM and analyzed using one-way ANOVA followed by Bonferroni’s post hoc test. * *p* < 0.05.

To determine whether the pharmacological inhibition of SMOX could translate into functional recovery following TBI in aged populations, we conducted a comprehensive battery of behavioral tests spanning motor, cognitive, and emotional domains. Behavioral performance was assessed seven days post-injury in aged mice treated with either JNJ-9350 or vehicle (Figure 5A).

Motor function was assessed using the Neurological Severity Score (NSS) and grip strength tests. Aged mice treated with vehicle displayed significant motor impairments following CCI, whereas JNJ-9350 (5 mg/kg) treatment markedly improved both NSSs and grip strength (Figure 5B), indicating enhanced motor recovery. Meanwhile, cognitive function was evaluated through the spontaneous Y-maze alternation test and the novel object recognition task. Vehicle-treated TBI mice exhibited pronounced deficits in both spatial working memory and recognition memory (Figure 5C). In contrast, SMOX inhibition significantly restored cognitive performance, as evidenced by improved spontaneous alternation percentages in the Y-maze and the increased exploration of novel objects in the NOR task (Figure 5C). Emotional behaviors were assessed by measuring center zone entries in the open-field test and latency to feed in the novelty-suppressed feeding test. Following CCI surgery, vehicle-treated aged mice demonstrated heightened anxiety-like behaviors, reflected by decreased center zone exploration and prolonged feeding latency (Figure 5D). SMOX inhibition by JNJ-9350 treatment effectively alleviated these anxiety-like behaviors, suggesting overall improvement in emotional regulation after injury (Figure 5D).

Taken together, these behavioral results demonstrate that the pharmacological inhibition of SMOX effectively reverses TBI-induced impairments across motor, cognitive, and emotional domains in aged mice. These findings strongly support the conclusion that age-associated SMOX upregulation contributes directly to oxidative stress-mediated neuronal dysfunction and increased vulnerability to TBI. Targeting SMOX therefore represents a promising therapeutic approach for improving outcomes in the aging brain after traumatic injury.

### 3.6. SMOX Expression Is Primarily Upregulated in Neurons and Microglia in the Aged Brain

To better understand the cellular mechanisms by which SMOX contributes to oxidative stress and the heightened susceptibility to traumatic brain injury observed in aged individuals, we then investigated the spatial and cell-type-specific expression of SMOX in the brain. While previous data demonstrated the robust upregulation of SMOX in the aged brain, the specific cell populations responsible for this increase remained unidentified. We hypothesized that distinct brain cell types may differentially express SMOX with aging, thereby contributing to cell-type-specific vulnerability and pathological responses following brain injury.

**Figure 6:**
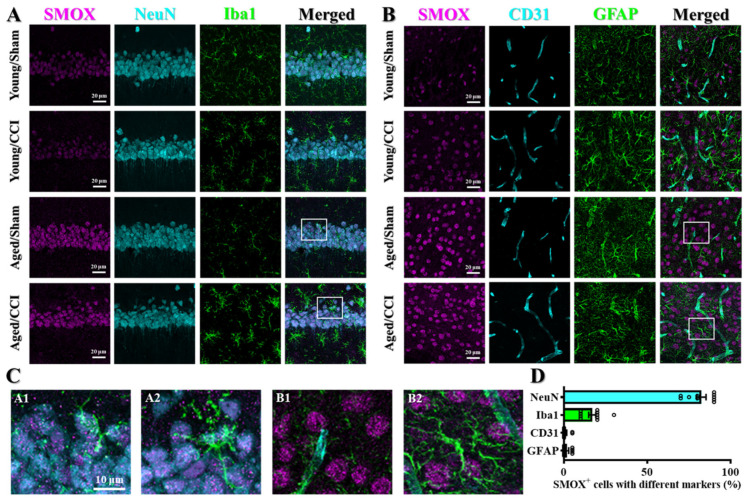
SMOX expression is primarily upregulated in neurons and microglia in aged brain. (**A**) Representative immunofluorescence images showing co-localization of SMOX (magenta) with NeuN (neuronal marker, cyan) and Iba1 (microglial marker, green) in hippocampal CA1 region. Scale bar = 20 μm. (**B**) Representative images showing co-staining of SMOX (magenta) with CD31 (endothelial marker, cyan) and GFAP (astrocytic marker, green) in hippocampal CA1 region. Scale bar = 20 μm. (**C**) High-magnification views of representative inset areas from (**A**,**B**), illustrating detailed cellular co-localization. Scale bar = 10 μm. (**D**) Quantification of percentage of SMOX-positive cells co-expressing cell-type-specific markers in aged mice. Data are presented as mean ± SEM.

To test this, we performed immunofluorescent staining on brain sections obtained from young and aged mice to visualize and quantify SMOX expression across major brain cell types (Figure 6A,B). SMOX-positive cells were co-stained with the established markers: we used NeuN for neurons, Iba1 for microglia, CD31 for cerebral endothelial cells, and GFAP for astrocytes. This approach enabled us to determine the relative contribution of each cell type to the elevated SMOX expression observed in the aged brain.

Our analysis revealed a striking pattern: SMOX expression in the aged brain was predominantly localized to NeuN-positive neurons, with a smaller subset of SMOX-positive cells co-expressing Iba1, indicative of microglial identity (Figure 6A,C). In contrast, we observed minimal colocalization of SMOX with CD31 or GFAP (Figure 6B,D), suggesting that endothelial cells and astrocytes do not significantly contribute to the SMOX upregulation seen with aging. These findings were consistently observed across multiple brain regions, including the cortex and hippocampus. Quantitative colocalization analysis further confirmed these observations. As shown in Figure 6D, approximately 82.5 ± 2.71% of all SMOX-positive cells were neurons, while the remaining 17.0 ± 2.13% were microglia. The proportions of SMOX-positive endothelial cells and astrocytes were negligible and did not show significant differences between young and aged animals (Figure 6D). This distribution pattern suggests a neuron- and microglia-specific role of SMOX in the aging brain.

Collectively, these results indicate that the age-associated increase in SMOX expression is primarily confined to neurons and, to a lesser extent, microglia. This cell-type-specific expression pattern suggests that SMOX may mediate pathological oxidative stress responses predominantly within these two populations. In particular, neurons may serve as the central site for SMOX-driven redox imbalance, while microglia may contribute to a secondary inflammatory amplification loop. These findings offer critical insight into how elevated SMOX expression might mechanistically increase vulnerability to brain injury in aging by impairing neuronal integrity and promoting microglial reactivity.

### 3.7. SMOX-Mediated Oxidative Stress Promotes Microglial Activation and Pro-Inflammatory Responses in the Aged Brain

**Figure 7:**
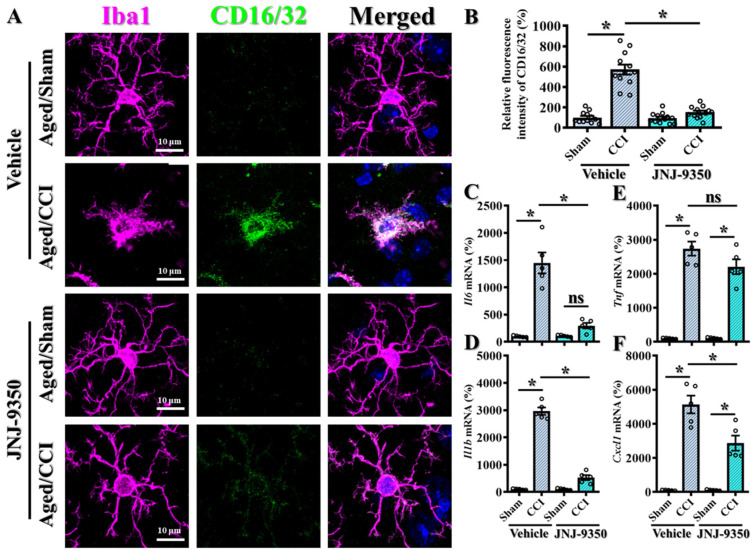
Pharmacological inhibition of SMOX reduces pro-inflammatory microglial activation in aged mice. (**A**) Representative immunofluorescence images showing co-staining of pro-inflammatory marker CD16/32 (green) and microglial marker Iba1 (magenta) in hippocampal CA1 region. Nuclei are counterstained with DAPI (blue). Scale bar = 10 μm. (**B**) Quantification of relative fluorescence intensity of CD16/32 across experimental groups. Quantification based on 12 images per group (n = 4 mice/group). (**C**–**F**) Quantitative analysis of mRNA expression levels of canonical pro-inflammatory mediators (*Il6*, *Il1b*, *Tnf*, and *Cxcl1*) in cortical tissue following CCI, with or without JNJ-9350 treatment. All data are presented as mean ± SEM and were analyzed using one-way ANOVA followed by Bonferroni’s post hoc test. * *p* < 0.05; ns, not significant.

Accumulating evidence suggests that elevated oxidative stress in microglial cells contributes to functional changes in these innate immune cells, shifting them toward a pro-inflammatory phenotype [34,35,36]. This transformation is believed to exacerbate neuro-inflammatory responses, particularly under pathological conditions such as traumatic brain injury. To investigate whether SMOX expression contributes to such pro-inflammatory microglial activation, we first examined the expression of CD16/32, a well-established marker of classically activated, pro-inflammatory microglia, in brain sections from animals subjected to CCI surgery (Figure 7A). Immunofluorescent co-staining for CD16/32 and Iba1 (a microglial marker) was used to identify activated microglia and assess their inflammatory status (Figure 7A).

Following CCI, we observed a pronounced morphological shift in microglial cells from a resting, ramified state to an activated, amoeboid morphology, indicative of profound microgliosis [37,38]. Notably, these morphologically reactive microglia exhibited markedly increased CD16/32 expression, indicating an enhanced pro-inflammatory phenotype (Figure 7A,B). This finding supports the notion that brain trauma triggers the activation of resident microglia and drives their polarization toward a pro-inflammatory state.

To determine whether SMOX upregulation contributes to microglial inflammatory activation, we assessed the effects of pharmacological SMOX inhibition using JNJ-9350. Aged mice were treated with either vehicle or JNJ-9350 prior to CCI surgery, and microglial activation and CD16/32 expression were subsequently analyzed (Figure 7A,B). Compared to vehicle-treated animals, JNJ-9350-treated mice exhibited a significant reduction in the relative fluorescent intensity of CD16/32-positive microglia (Figure 7 B; JNJ-9350/CCI: 151.5 ± 16.75% vs. vehicle/CCI: 571.3 ± 49.08%, *p* < 0.01). Moreover, microglia in JNJ-treated brains retained a predominantly ramified, quiescent morphology, indicating the suppression of both morphological and functional activation (Figure 7A). These findings suggest that SMOX activity plays a key role in driving both morphological and functional activation of microglia following brain injury.

In parallel, we analyzed the mRNA expression levels of canonical pro-inflammatory mediators—*Il6*, *Il1b*, *Tnf*, and *Cxcl1*—in cortical tissue following CCI. As expected, TBI induced the significant upregulation of these cytokines and chemokines (Figure 7C–F). Notably, JNJ-9350 treatment significantly reduced the expression of *Il6*, *Il1b*, and *Cxcl1*, while the decrease in *Tnf* expression showed a non-significant downward trend. Together, these data further support the central role of SMOX in promoting pro-inflammatory responses in the aged brain following traumatic brain injury.

Collectively, these findings demonstrate that SMOX plays a pivotal role in promoting microglial activation and amplifying neuro-inflammation following TBI. The age-associated upregulation of SMOX in microglia appears to mediate oxidative stress-induced pro-inflammatory changes that exacerbate TBI pathology. Notably, the pharmacological inhibition of SMOX effectively suppressed microglial activation and reduced the expression of key pro-inflammatory mediators, highlighting SMOX as a promising therapeutic target for mitigating age-related vulnerability to brain injury.

### 3.8. SMOX Inhibition Attenuates Oxidative Stress-Induced Neuronal Degeneration and Apoptotic Signaling in the Aged Brain

**Figure 8:**
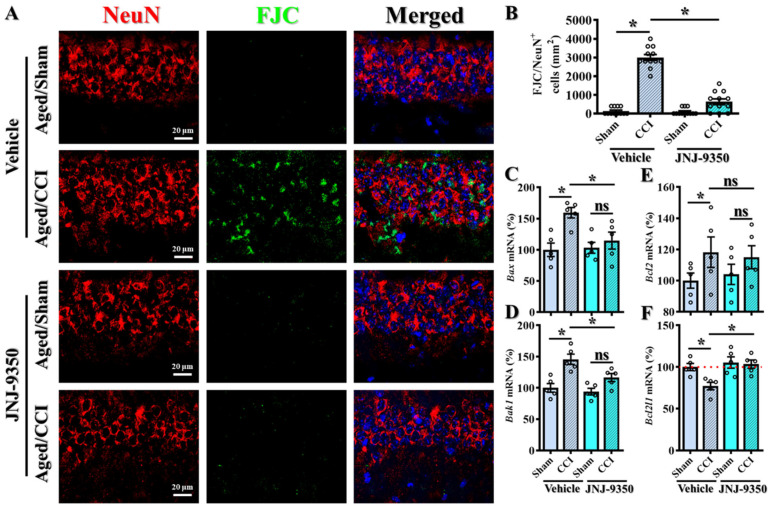
Pharmacological inhibition of SMOX reduces neuronal degeneration and apoptotic signaling in the aged brain following TBI. (**A**) Representative immunofluorescence images showing co-staining of degenerating neurons with Fluoro-Jade C (FJC, green) and neuronal marker NeuN (red) in hippocampal CA1 region. Nuclei are counterstained with DAPI (blue). Scale bar = 20 μm. (**B**) Quantification of FJC-positive neurons across experimental groups. Quantification based on 12 images per group (n = 4 mice/group). (**C**–**F**) Quantitative real-time PCR analysis of mRNA expression levels of pro-apoptotic genes (*Bax* and *Bak1*) and anti-apoptotic genes (*Bcl2* and *Bcl2l1*) in cortical tissue following CCI, with or without JNJ-9350 treatment. Small circles in B and C–F represent individual data points from biological replicates. All data are presented as mean ± SEM and analyzed using one-way ANOVA followed by Bonferroni’s post hoc test. * *p* < 0.05; ns, not significant.

Extensive evidence has established the existence of a strong link between oxidative stress and neuronal vulnerability, where excessive reactive oxygen species (ROS) drive oxidative damage, impair neuronal function, and ultimately lead to neuronal cell death [39,40,41]. Given our earlier findings that SMOX is predominantly upregulated in neurons in the aged brain, we next investigated whether the increased SMOX contributes to neuronal degeneration following traumatic brain injury and whether its pharmacological inhibition could confer neuroprotection (Figure 8A).

To assess neuronal degenerating cell death, we employed Fluoro-Jade C (FJC) staining in combination with the neuronal marker NeuN to identify degenerating neurons in brain tissue collected from aged mice subjected to CCI surgery. As expected, CCI induced a substantial increase in FJC-positive degenerating neurons in aged animals, consistent with widespread neuronal injury (Figure 8A,B). Importantly, treatment with the SMOX inhibitor JNJ-9350 significantly reduced the number of FJC/NeuN double-positive cells (Figure 8B; JNJ-9350/CCI: 633.3 ± 151.4 cells/mm^2^ vs. vehicle/CCI: 3000 ± 159.5 cells/mm^2^, *p* < 0.01), indicating a strong protective effect against injury-induced neuronal degeneration.

To further elucidate the molecular mechanisms underlying SMOX-driven neuronal vulnerability, we analyzed the expression of key apoptotic regulators using real-time PCR. Specifically, we measured the mRNA levels of pro-apoptotic genes (*Bax* and *Bak1*) and anti-apoptotic genes (*Bcl2* and *Bcl2l1*) in brain tissue from aged mice following CCI, with or without SMOX inhibition (Figure 8C–F). Our results showed that CCI significantly upregulated *Bax* expression while downregulating *Bcl2l1*, indicating a shift toward a pro-apoptotic transcriptional profile. Notably, SMOX inhibition with JNJ-9350 effectively reversed these alterations, restoring *Bcl2l1* expression and reducing *Bax* levels (Figure 8C–F). Bak1 expression also trended toward normalization, although the change did not reach the level of statistical significance.

Collectively, these findings strongly suggest that the age-dependent upregulation of SMOX in neurons exacerbates oxidative stress and contributes to neuronal degeneration through the activation of apoptotic pathways. The pharmacological inhibition of SMOX not only reduced the extent of neuronal degeneration, as demonstrated by FJC staining, but also corrected the dysregulated expression of apoptosis-related genes. These results underscore the therapeutic potential of targeting SMOX to protect neurons from oxidative damage and cell death in the aged brain following TBI.

### 3.9. SMOX Inhibition Preserves GLT-1 Expression and Astrocytic Glutamate Uptake Capacity in the Aged Brain Following TBI

Although our current study emphasizes neuronal and microglial SMOX expression, emerging evidence from SMOX-overexpressing transgenic mice has demonstrated that elevated SMOX levels can impair astrocytic glutamate uptake and promote glutamate efflux via the cystine/glutamate antiporter system xc^−^, contributing to excitotoxic injury [42,43]. To examine whether similar mechanisms are relevant in the aged brain following TBI, we evaluated the expression and localization of key glutamate transporters in our model.

**Figure 9:**
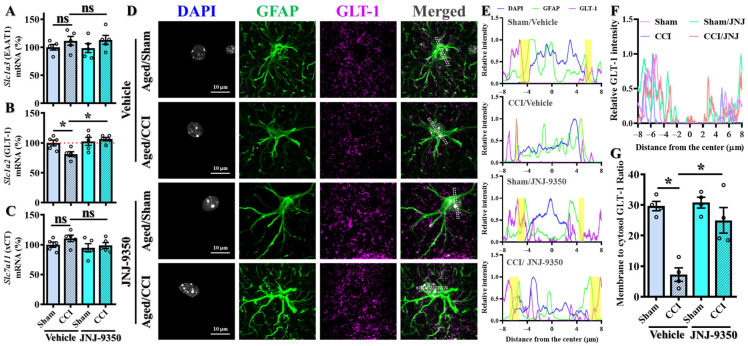
SMOX inhibition restores GLT-1 expression and astrocytic transporter localization in aged brain following TBI. (**A**–**C**) Quantitative PCR analysis of glutamate transporter gene expression in cortex of aged mice across experimental groups. CCI significantly decreased Slc1a2 (GLT-1) mRNA expression, which was reversed by SMOX inhibition (**B**), while Slc1a3 (EAAT1) (**A**) and SLC7A11 (xCT) (**C**) levels remained unchanged. (**D**) Representative immunofluorescence images showing co-localization of GFAP (green) and GLT-1 (magenta) in cortical astrocytes. Nuclei are counterstained with DAPI (blue). Scale bar = 10 μm. Dashed lines in merged images indicate regions used for line-plot quantification. (**E**) Line-plot analysis of GLT-1 fluorescence intensity across astrocytic regions from (**D**). Yellow-shaded areas represent membrane regions used to calculate membrane-to-cytosol ratios. (**F**) Quantification of GLT-1 signal in GFAP-positive astrocytes across experimental groups. (**G**) Quantification of membrane-to-cytosol GLT-1 ratio in astrocytes. CCI significantly reduced astrocytic membrane-localized GLT-1 in aged mice, effect reversed by JNJ-9350 treatment. Data are presented as mean ± SEM and were analyzed using one-way ANOVA followed by Bonferroni’s post hoc test. * *p* < 0.05.

Quantitative PCR analysis revealed a significant reduction in Slc1a2 (GLT-1) expression in the cortex of aged mice following CCI, whereas JNJ-9350 treatment effectively restored GLT-1 levels to near baseline levels (Figure 9B). In contrast, expression levels of Slc1a3 (EAAT1) and SLC7A11 (xCT) remained unchanged across experimental groups (Figure 9A,C), suggesting that GLT-1 is selectively susceptible to injury- and SMOX-related modulation in the aged brain.

To assess whether these transcriptional changes were reflected at the protein level in astrocytes, we performed double immunofluorescent staining for GLT-1 (also known as Excitatory Amino Acid Transporter 2, EAAT2) and the astrocytic marker GFAP. While total GLT-1 intensity in GFAP-positive cells was only modestly reduced after CCI (Figure 9D–F), the membrane-to-cytosol ratio of GLT-1 signal was significantly decreased, indicating functional impairment in transporter localization (Figure 9G). Importantly, this deficit was rescued by JNJ-9350 treatment, supporting the idea that SMOX inhibition restores membrane-bound GLT-1 and thus preserves astrocytic glutamate uptake capacity.

These findings indicate that while SMOX is not predominantly expressed in astrocytes, its pathological upregulation in the aged brain indirectly impairs astrocytic glutamate clearance capacity. The resulting dysregulation of glutamate homeostasis may contribute to excitotoxic neuronal injury, and can be effectively mitigated by pharmacological SMOX inhibition.

### 3.10. SMOX Inhibition Confers Long-Term Functional and Neuroprotective Benefits Following Traumatic Brain Injury in Aged Mice

**Figure 10:**
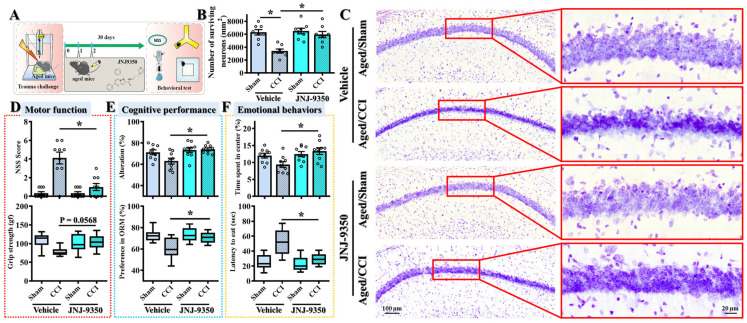
SMOX inhibition with JNJ-9350 enhances long-term recovery from TBI in aged mice. (**A**) Schematic overview of experimental design used to assess long-term effects of SMOX inhibition on functional and histological outcomes in aged mice following CCI. (**B**,**C**) Quantification (**B**) and representative images (**C**) of Nissl-positive surviving neurons in the hippocampal CA1 region 30 days after CCI surgery, with or without JNJ-9350 treatment. (**D**) Quantification of motor function, assessed by Neurological Severity Score (NSS) and grip strength testing, in vehicle- and JNJ-9350-treated mice. (**E**) Cognitive performance measured by Y-maze spontaneous alternation test and novel object recognition memory (ORM) test. (**F**) Emotional behavior assessed through open-field center zone exploration and latency to feed in novelty-suppressed feeding (NSF) test. All data are presented as mean ± SEM and were analyzed using one-way ANOVA followed by Bonferroni’s post hoc test. * *p* < 0.05.

While our earlier findings demonstrated that the pharmacological inhibition of SMOX mitigates acute injury responses and improves short-term outcomes at seven days post-TBI, a critical consideration for clinical translation is whether such treatment can confer sustained functional benefits during long-term recovery. Given the chronic nature of TBI-related disabilities, particularly in aged individuals who exhibit heightened susceptibility, we evaluated the lasting impact of SMOX inhibition on behavioral and histological outcomes at thirty days following injury (Figure 10A).

To address this, aged mice subjected to CCI surgery were treated with the SMOX inhibitor JNJ-9350 for three consecutive days following the challenge. Behavioral assessments of motor, cognitive, and emotional function were conducted thirty days post-injury using a standardized test battery. As expected, vehicle-treated mice exhibited persistent impairments across multiple domains (Figure 10A,D–F), including motor coordination deficits, spatial learning and memory dysfunction, and elevated anxiety-like behaviors—hallmarks of long-term TBI pathology. Remarkably, JNJ-9350-treated mice demonstrated significantly improved performance in all tested domains, indicating that the short-term pharmacological inhibition of SMOX confers enduring therapeutic effects and promotes long-term functional recovery in the aged brain (Figure 10D–F).

To further validate the long-term neuroprotective effects of SMOX inhibition, we performed Nissl staining to assess neuronal survival in the hippocampal CA1 region—a brain area particularly vulnerable to TBI-induced neurodegeneration. Consistent with the observed behavioral impairments, aged mice exhibited substantial neuronal loss in the CA1 region thirty days after the CCI challenge (Figure 10B–C). Notably, treatment with JNJ-9350 significantly increased the number of surviving neurons in this region, indicating that SMOX inhibition not only enhances functional outcomes but also preserves neuronal integrity during the chronic phase of recovery.

Together, these results strongly support the hypothesis that the age-associated upregulation of SMOX contributes to sustained neurodegeneration and functional decline following TBI. Importantly, the transient inhibition of SMOX in the early post-injury period exerts long-lasting protective effects on both neuronal survival and behavioral performance. These findings highlight the translational potential of SMOX inhibition as a viable therapeutic strategy to improve long-term outcomes in aged, TBI-susceptible individuals.

## 4. Discussion

The present study provides compelling evidence that the age-dependent upregulation of spermine oxidase (SMOX) plays a critical role in modulating oxidative stress, neuro-inflammation, and neuronal vulnerability following traumatic brain injury. By integrating cell-type-specific analyses, molecular profiling, and both short- and long-term behavioral assessments, we demonstrated that increased SMOX expression in the aged brain contributes to a heightened susceptibility to TBI. Moreover, the pharmacological inhibition of SMOX using JNJ-9350 yields significant neuroprotective and functional benefits, highlighting its potential as a therapeutic target.

While SMOX has been previously reported to be inducible after TBI, notably by Zahedi and colleagues who showed significant SMOX upregulation in both neurons and glia post-injury in adult rats [20], our findings add an important age-related dimension. In young adult mice, we confirmed that SMOX expression is increased following CCI, particularly in brain regions proximal to the impact site such as the somatosensory and motor cortices (Supplementary Figure S1). In contrast, aged mice displayed a markedly elevated basal level of SMOX in these same regions, with no further increase after injury. This pattern suggests that in the aged brain, SMOX expression may already be pathologically saturated, potentially limiting the capacity for further induction following trauma. These observations reconcile prior reports of injury-induced SMOX expression with our findings by highlighting that aging and TBI may converge on the same oxidative pathway, but with differing regulatory thresholds. This constitutive SMOX upregulation in aging may thus serve as a predisposing factor that amplifies TBI vulnerability by shifting the redox balance toward a more reactive state even before injury occurs.

A major finding of this study is the distinct cellular distribution pattern of SMOX expression, which was primarily localized to neurons and, to a lesser extent, microglia in aged animals. This distribution suggests the dual contribution of SMOX to TBI pathology: intrinsic neuronal vulnerability and extrinsic inflammatory amplification. Neurons, known for their sensitivity to redox imbalance, appear to be principal targets of SMOX-driven oxidative stress. Microglial cells, meanwhile, showed partial colocalization with SMOX and responded to injury with an exaggerated inflammatory phenotype, marked by morphological activation and the increased expression of CD16/32. These observations align with previous reports indicating that oxidative stress not only causes direct neuronal damage but also primes glial cells toward a reactive, pro-inflammatory phenotype that further exacerbates neurodegeneration [36,44].

Another significant contribution of this work is the demonstration that short-term inhibition of SMOX confers long-term neuroprotective benefits. While many preclinical TBI studies emphasize acute or subacute time points [45], our behavioral and histological data at 30 days post-injury provide compelling evidence that early intervention targeting SMOX can mitigate chronic functional decline and neuronal loss. This finding carries important translational relevance, particularly for aged individuals, who are not only more vulnerable to TBI but also prone to prolonged recovery and poorer long-term outcomes. The ability of JNJ-9350 to improve motor, cognitive, and emotional outcomes, along with enhanced neuronal preservation in the hippocampus, supports the feasibility of SMOX as a therapeutic target for long-term neuroprotection in the aging brain.

While SMOX was not highly expressed in astrocytes in our model, our data suggest that its upregulation in neurons and microglia may indirectly impair astrocyte function. Previous reports have shown that SMOX overexpression in transgenic mice leads to impaired glutamate uptake and enhanced glutamate efflux via system xc^−^, contributing to excitotoxicity [42]. In line with these findings, we observed that CCI significantly decreased GLT-1 expression and disrupted its membrane localization in astrocytes from aged mice, effects that were reversed by SMOX inhibition with JNJ-9350. Interestingly, EAAT1 and xCT levels remained unchanged, suggesting the selective vulnerability of GLT-1 to SMOX-related oxidative stress. These results implicate SMOX as an upstream regulator of impaired glutamate homeostasis, whereby its pathological activation in the aged brain may exacerbate excitotoxic injury through astrocytic dysfunction, even in the absence of direct SMOX induction in astrocytes.

A key strength of this study is the identification of SMOX as a selective therapeutic target that specifically addresses age-associated TBI pathology. Unlike general anti-inflammatory or anti-oxidant strategies that often lack cell-type specificity and show limited efficacy in aged individuals, targeting SMOX directly could interrupt the upstream source of oxidative damage that disproportionately affects neurons and microglia in the aged brain. This mechanistically guided approach offers a path toward developing interventions that are tailored to the unique pathophysiological features of TBI in older populations, who have been historically underrepresented in preclinical and clinical research.

Moreover, the pathological role of SMOX may extend beyond TBI. Emerging evidence suggests that SMOX functions as a common pathological driver across multiple age-related conditions, including neurodegenerative diseases, cardiovascular disorders, and metabolic dysfunctions, all of which share underlying features of chronic oxidative stress and inflammation. The findings of this study not only highlight SMOX’s contribution to brain injury but also raise the broader possibility that it serves as an aging-associated mediator of cellular stress. Thus, SMOX could represent a converging molecular node in the pathogenesis of diverse age-related diseases and deserves further investigation as a systemic therapeutic target.

Despite the promising results observed with JNJ-9350, there remains a clear need for the development of next-generation SMOX inhibitors with enhanced potency and central nervous system (CNS) bioavailability. Although the current compound effectively reduced injury severity and improved long-term outcomes, further optimization of its pharmacokinetic properties—including blood–brain barrier permeability, metabolic stability, and dosing flexibility—is essential for later clinical translation. Future SMOX inhibitors should be specifically designed for efficient CNS delivery and validated in both acute and delayed treatment paradigms. Such improvements will be critical for accelerating the path toward clinical trials and ultimately integrating SMOX-targeted therapies into standard-of-care strategies for TBI, particularly in aged populations.

While our findings are encouraging, several limitations should be acknowledged. First, the study was conducted exclusively in aged male mice (36–40 weeks old); therefore, potential sex-specific differences in SMOX expression and therapeutic response remain unexplored and warrant further investigation. Second, although JNJ-9350 is a selective SMOX inhibitor, its pharmacokinetic profile and optimal dosing parameters for clinical use are not yet fully defined. Future studies should also evaluate delayed treatment paradigms to better simulate clinical scenarios and assess the therapeutic window for effective SMOX inhibition beyond the acute injury phase.

## 5. Conclusions

**Figure 11:**
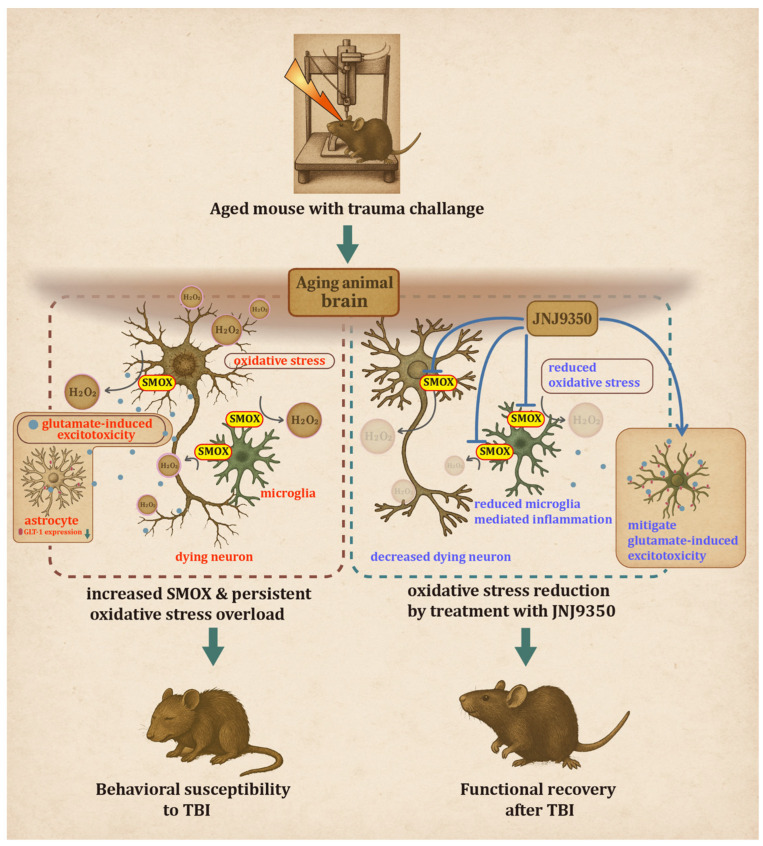
Cartoon illustration summarizing key findings of current study. In aged brain, SMOX is pathologically upregulated in neurons and microglia, leading to increased oxidative stress, neuro-inflammation, and neuronal degeneration following traumatic brain injury. In addition, SMOX dysregulation indirectly impairs astrocytic glutamate uptake by disrupting GLT-1 membrane localization, contributing to excitotoxicity. Pharmacological inhibition of SMOX using JNJ-9350 attenuates these pathological processes, resulting in reduced microglial activation, preserved neuronal integrity, and improved motor, cognitive, and emotional outcomes. These findings position SMOX as therapeutic target for mitigating age-related vulnerability to TBI.

Our study underscores the pathological role of SMOX in exacerbating TBI outcomes in the aged brain. By linking SMOX activity to oxidative stress, neuro-inflammation, and neuronal loss, we demonstrate that its inhibition can meaningfully improve both functional and histological outcomes (Figure 11). Notably, our findings also reveal that SMOX dysregulation indirectly impairs astrocytic glutamate transporter function, contributing to excitotoxic injury, an effect reversible by SMOX inhibition. These results not only enhance our understanding of the molecular mechanisms underlying age-related TBI vulnerability but also establish SMOX as a modifiable and disease-relevant therapeutic target. Moving forward, addressing the pharmacological limitations of current SMOX inhibitors will be critical for translating this strategy into clinically viable interventions for aged individuals at heightened risk of TBI.

## Figures and Tables

**Table 1:** The list of the primer pairs used in the current study.

Primer	Accession Number	Forward (Sense)	Reverse (Antisense)
**Antioxidant-related genes**			
*Cybb* (NOX2)	NM_007807	TGGCGATCTCAGCAAAAGGTGG	GTACTGTCCCACCTCCATCTTG
*Paox*	NM_153783	CTCCCTGAAGATGGAACTGGCA	GACTAACGAGGCGATCAGCTTC
*Smox*	NM_145533	TGCTACCTTACCAACCGTGGCT	ACGCTGTTCTGACTCTCGGCAT
*Xdh*	NM_011723	GCTCTTCGTGAGCACACAGAAC	CCACCCATTCTTTTCACTCGGAC
*Maob*	NM_172778	TACTTGGGGACCGAGTGAAGCT	CCAAAGCAGGTGGAATGGCACT
**Inflammatory-related genes**			
*Tnf*	NM_013693	GGTGCCTATGTCTCAGCCTCTT	GCCATAGAACTGATGAGAGGGAG
*Il-6*	NM_031168	TACCACTTCACAAGTCGGAGGC	CTGCAAGTGCATCATCGTTGTTC
*Il-1β*	NM_008361	TGGACCTTCCAGGATGAGGACA	GTTCATCTCGGAGCCTGTAGTG
*Cxcl1*	NM_008176	TCCAGAGCTTGAAGGTGTTGCC	AACCAAGGGAGCTTCAGGGTCA
**Cell death-related genes**			
Bcl-2	NM_009741	CCTGTGGATGACTGAGTACCTG	AGCCAGGAGAAATCAAACAGAGG
Bcl2l1	NM_009743	GCCACCTATCTGAATGACCACC	AGGAACCAGCGGTTGAAGCGC
Bax	NM_007527	AGGATGCGTCCACCAAGAAGCT	TCCGTGTCCACGTCAGCAATCA
Bak1	NM_007523	GGAATGCCTACGAACTCTTCACC	CAAACCACGCTGGTAGACGTAC
**Glutamate transporters**			
*Slc1a3* (EAAT1)	NM_148938	GCGATTGGTCGCGGTGATAATG	CGACAATGACTGTCACGGTGTAC
*Slc1a2* (GLT-1)	NM_001077514	TTCCAAGCCTGGATCACTGCTC	GGACGAATCTGGTCACACGCTT
*Slc7a11* (xCT)	NM_011990	CTTTGTTGCCCTCTCCTGCTTC	CAGAGGAGTGTGCTTGTGGACA
**Internal controls**			
*Gapdh*	NM_008084	CATCACTGCCACCCAGAAGACTG	ATGCCAGTGAGCTTCCCGTTCAG

## Data Availability

All data are available on request from the corresponding author.

## Supplementary Materials

The following supporting information can be downloaded at: https://www.mdpi.com/article/10.3390/antiox14060709/s1, Figure S1: Increased SMOX expression following CCI in the somatosensory and motor cortices of young but not aged mice.
