# Supplementary material for: Targeting Spermine Oxidase to Mitigate Traumatic Brain Injury Pathology in the Aging Brain
Source: Antioxidants (Basel). 2025 Jun 11;14(6):709. doi: 10.3390/antiox14060709 (PMC12189194; doi:10.3390/antiox14060709)
Supplement: Supplementary file 1 [file antioxidants-14-00709-s001.zip › antioxidants-3649208-supplementary.pptx]

## Slide 1
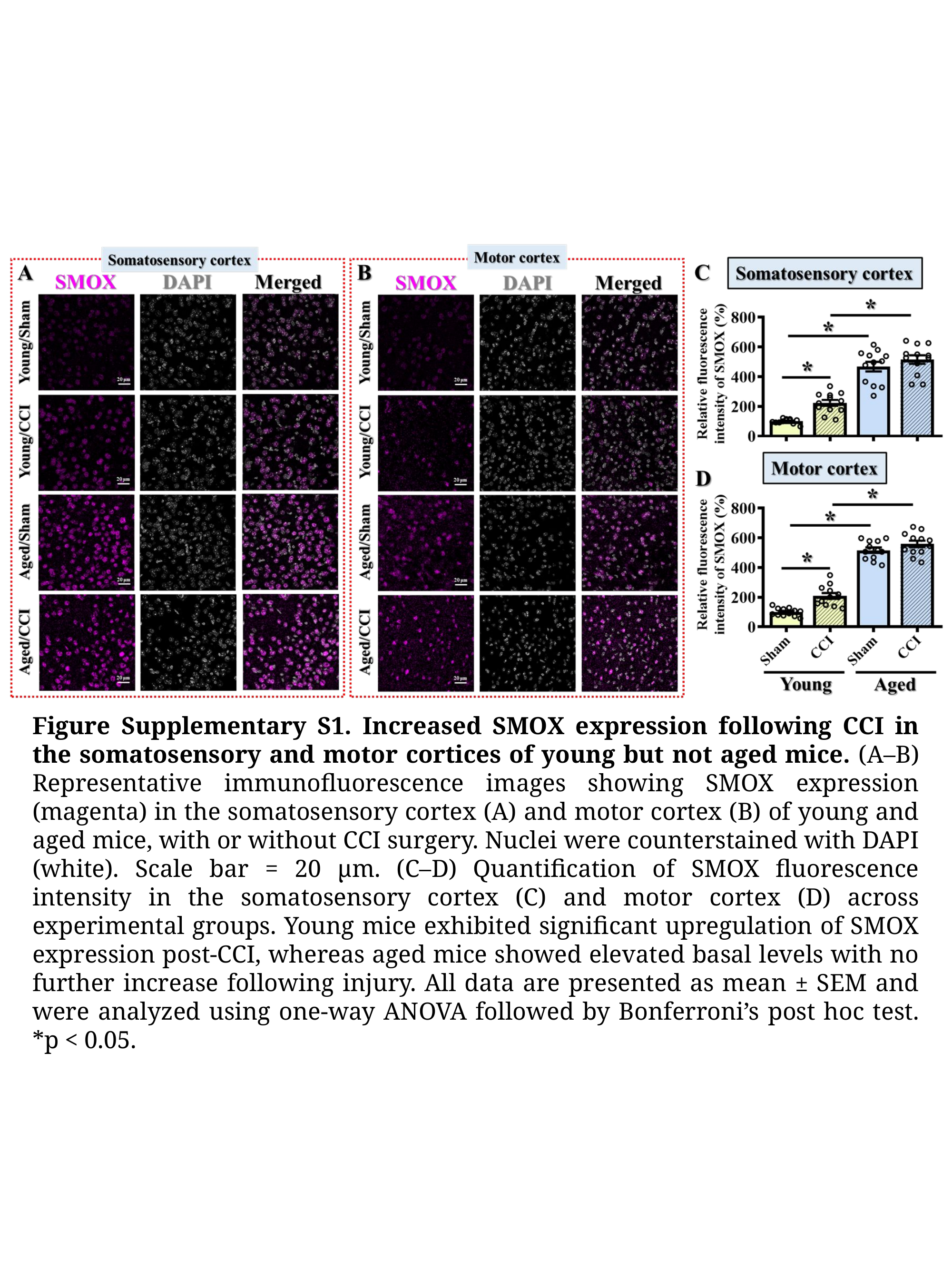

Figure Supplementary S1. Increased SMOX expression following CCI in the somatosensory and motor cortices of young but not aged mice. (A–B) Representative immunofluorescence images showing SMOX expression (magenta) in the somatosensory cortex (A) and motor cortex (B) of young and aged mice, with or without CCI surgery. Nuclei were counterstained with DAPI (white). Scale bar = 20 μm. (C–D) Quantification of SMOX fluorescence intensity in the somatosensory cortex (C) and motor cortex (D) across experimental groups. Young mice exhibited significant upregulation of SMOX expression post-CCI, whereas aged mice showed elevated basal levels with no further increase following injury. All data are presented as mean ± SEM and were analyzed using one-way ANOVA followed by Bonferroni’s post hoc test. *p < 0.05.
